# Comparison of barriers to effective nurse-patient communication in COVID-19 and non-COVID-19 wards

**DOI:** 10.1186/s12912-024-01947-4

**Published:** 2024-05-16

**Authors:** Hamed Bakhshi, Mohammad Javad Shariati, Mohammad Hasan Basirinezhad, Hossein Ebrahimi

**Affiliations:** 1 Student Research Committee, School of Nursing and Midwifery, Shahroud University of Medical Sciences, Shahroud, Iran; 2 Department of Epidemiology and Biostatistics, School of Public Health, Shahid Sadoughi University of Medical Sciences, Yazd, Iran; 3 Center for Health Related Social and Behavioral Sciences Research, Shahroud University of Medical Sciences, Shahroud, Iran

**Keywords:** Communication barriers, Communication, Nurse–patient communication, Nurses, Patients

## Abstract

**Background:**

Communication is a basic need of humans. Identifying factors that prevent effective nurse-patient communication allows for the better implementation of necessary measures to modify barriers. This study aims to compare the barriers to effective nurse-patient communication from the perspectives of nurses and patients in COVID-19 and non-COVID-19 wards.

**Materials and methods:**

This cross-sectional descriptive study was conducted in 2022. The participants included 200 nurses (by stratified sampling method) and 200 patients (by systematic random sampling) referred to two conveniently selected hospitals in Shahroud, Iran. The inclusion criteria for nurses were considered having at least a bachelor’s degree and a minimum literacy level for patients to complete the questionnaires. Data were collected by the demographic information form and questionnaire with 30 and 15 questions for nurses and patients, which contained similar questions to those for nurses, based on a 5-point Likert scale. Data were analysis using descriptive indices and inferential statistics (Linear regression) in SPSS software version 18.

**Results:**

The high workload of nursing, excessive expectations of patients, and the difficulty of nursing work were identified by nurses as the main communication barriers. From the patients’ viewpoints, the aggressiveness of nurses, the lack of facilities (welfare treatment), and the unsanitary conditions of their rooms were the main communication barriers. The regression model revealed that the mean score of barriers to communication among nurses would decrease to 0.48 for each unit of age increase. Additionally, the patient’s residence explained 2.3% of the nurses’ barriers to communication, meaning that native participants obtained a mean score of 2.83 units less than non-native nurses, and there was no statistically significant difference between the COVID and non-COVID wards.

**Conclusion:**

In this study, the domain of job characteristics was identified by nurses as the major barrier, and patients emphasized factors that were in the domain of individual/social factors. There is a pressing need to pay attention to these barriers to eliminate them through necessary measures by nursing administrators.

## Background

First observed in Wuhan, China, the COVID-19 pandemic is an acute and very severe respiratory syndrome that the World Health Organization has raised as a health problem because of its high spread rate and consequences on an international scale. The number of COVID-19 patients is increasingly on the rise [1, 2]. Illness and hospitalization are usually stressful and associated with bad experiences for patients and their family members [3].

According to Tabandeh Sadeghi et al. (2011) “Communication is a basic need of humans. Any interaction is an opportunity to achieve effective communication and participation in understanding the issue, which leads to the achievement of mutual goals by individuals.” [4]. The three important aspects of communication that are emphasized the most are the message’s sender, the receiver, and the environment. Communicating is an interaction between the sender and the receiver of the message, and the environment affects them [5, 6]. In the context of a hospital, these three aspects of communication can be defined as nurse, patient, and hospital environment, and all three should be considered when examining the obstacles [7]. “According to Ali Fakhr Movahedi et al. (2012)” Communication is considered a central concept in nursing and an essential part of nursing work [8]. Patients perceive interaction with nurses as the basis of their treatment [9]. Nurse-patient communication is an interpersonal process that is created between these two groups during treatment. This process generally includes the start, work, and end stages. Effective communication is an essential aspect of patient care by nurses, and many nursing tasks cannot be performed without this activity [10]. Effective communication consists of explicit transmission and receipt of message content, in which information is consciously and unconsciously produced by a person and communicated to the recipient through verbal and non-verbal patterns [11]. The non-verbal aspect of communication plays an essential role and is more important than the verbal aspect of language in emergencies. The mandatory use of face masks during the COVID-19 pandemic negatively influenced nurse-patient communication, notably because this tool significantly reduced the messages arising from non-verbal communication channels [12]. In this regard, Vitale et al. investigated wearing face masks as a communication barrier between nurses and patients. The results showed no differences in the patients’ opinions before and during the COVID-19 pandemic; patients believed that the mask was not a communication barrier, while nurses thought that wearing masks was a communication barrier [12]. Unfavorable communication can hamper the patient’s recovery and may even permanently deprive the patient of health or life.

In comparison, good communication affects the patient’s recovery more than medication. In fact, nurses will succeed in their tasks when they can communicate well with their patients [13]. Effective communication can affect pain control, adherence to a treatment regimen, and the patient’s mental health and play an important role in reducing the patient’s anxiety and fear and faster recovery [14]. During good communication, patients can disclose and express sensitive and personal information. Consequently, nurses can also transfer necessary information, attitudes, or skills [4]. Identifying factors that prevent effective nurse-patient communication allows for the better implementation of measures required to adjust obstacles [15].

The first published reports of the deaths of coronavirus-infected doctors during caring for patients indicate that the virus transmission to healthcare workers in healthcare centers is a hazardous issue [16, 17]. Under these stressful conditions, nurses must manage long shift hours and the fear of contagion and overcome communication difficulties through layers of personal protective equipment. These problems may disrupt communication with patients and cause less focus of health workers on the psychosocial well-being of patients [18, 19]. Baillie states that the lack of time is a clear barrier to communication between emergency nurses and patients [20]. Meehan et al. also reported that nurses mentioned the lack of time, fatigue, and workload of personnel to be the factors preventing nurse-patient interaction. In the same research, patients cited the issue of gender as a factor preventing their interaction with nurses. However, male and female patients had difficulty communicating with male nurses [21].

Identifying factors that prevent effective nurse-patient communication makes it possible to elucidate the direction of necessary measures for the planners and executives of the health sector to eliminate or modify barriers. In particular, when these barriers are identified and expressed with a realistic approach, i.e., from nurses’ and patients’ perspectives [22]. Before this, no study compared barriers to nurse-patient communication in COVID and non-COVID wards. Therefore, this research aims to compare the barriers to effective nurse-patient communication from nurses’ and patients’ perspectives in COVID-19 and NON-COVID-19 wards. Hopefully, identifying these obstacles and planning to solve them as soon as possible will make us have nurses in the future who can communicate well with patients and improve service delivery.

## Materials and methods

### Study design

This cross-sectional descriptive research was conducted on 200 nurses and 200 patients at hospitals affiliated with the Shahroud University of Medical Sciences. The participants included nurses and patients from different wards of two conveniently selected hospitals in Shahroud. To sample nurses by the stratified method, the sample size was first divided by the total number of nurses in the mentioned hospitals to obtain the sampling fraction. According to Mohammadi et al. study, standard deviations reported for all subscales for barrier’s to effective communication (individual/social factors = 6.22), job characteristics = 6.74, patient’s clinical conditions = 4.22), and environmental factors = 9.09) were utilized to estimate the sample size [23]. Estimation error was considered 0.15 of standard deviation values. The confidence levels and power were considered at 0.95 and 0.8 respectively with a 15% dropout probability. Also, another sample size was calculated similarly using the standard deviation reported in Norouzinia et al. study for patient’s questionnaire equal to 1.96 [24]. Finally, among the estimated values; the largest number (200) was considered as the sample size of the present study for nurses and patients.

$$\begin{array}{l}{\rm{Individual}}\,{\rm{Social}}\,{\rm{Factors}}\,{\rm{N = }}\frac{{z_{1 - \frac{a}{2}}^2{\sigma ^2}}}{{{d^2}}}\\= \frac{{{{(1.96)}^2}{{(6.22)}^2}}}{{{{(0.99)}^2}}} = 172\end{array}$$

$$\begin{array}{l}{\rm{Job}}\,{\rm{Characteristics}}\,{\rm{Factors}}\,{\rm{N = }}\frac{{z_{1 - \frac{a}{2}}^2{\sigma ^2}}}{{{d^2}}}\\= \frac{{{{(1.96)}^2}{{(6.74)}^2}}}{{{{(1.0)}^2}}} = 175\end{array}$$

$$\begin{array}{l}{\rm{Patient's}}\,{\rm{Clinical}}\,{\rm{Conditions}}\,{\rm{N = }}\frac{{z_{1 - \frac{a}{2}}^2{\sigma ^2}}}{{{d^2}}}\\= \frac{{{{(1.96)}^2}{{(4.22)}^2}}}{{{{(0.63)}^2}}} = 173\end{array}$$

$$\begin{array}{l}{\rm{Environmental}}\,{\rm{Factors}}\,{\rm{N = }}\frac{{z_{1 - \frac{a}{2}}^2{\sigma ^2}}}{{{a^2}}}\\= \frac{{{{(1.96)}^2}{{(9.09)}^2}}}{{{{(1.36)}^2}}} = 172\end{array}$$

$$\begin{array}{l}{\rm{Patient's}}\,{\rm{questionnaire}}\,{\rm{N = }}\frac{{z_{1 - \frac{a}{2}}^2{\sigma ^2}}}{{{d^2}}}\\= \frac{{{{(1.96)}^2}{{(1.96)}^2}}}{{{{(0.294)}^2}}} = 171\end{array}$$

Considering that the total number of nurses is around 700 and the sample size calculated by the statistics consultant is 200 nurses, our sampling fraction was calculated as $\frac{2}{7}$. Therefore, $\frac{2}{7}$ personnel of each department were included in the study. The patients were sampled by a systematically random method using the hospital list, file number, and dates of admission and discharge. The inclusion criteria for nurses were a bachelor’s degree or higher and a minimum literacy level for patients to complete the questionnaire. Moreover, the questionnaire contained questions about the nurses’ work experience or no experience in COVID-19 wards. The duration of working in COVID-19 wards was included in the questionnaire questions, and the duration was considered in the analysis. Data were collected using a questionnaire provided to the nurses through daily visits to various wards of the mentioned hospitals, including emergency, surgery, special care, internal medicine, gastroenterology, cardiology, urology, orthopedics, ICU, CCU, and other wards. The questionnaire was also provided to the patients hospitalized in surgery, special care, internal medicine, gastroenterology, cardiology, urology, ICU, and CCU wards, among others. Due to the reduced coronavirus spread during that period, the information on COVID-19 patients was accessed using hospital information by obtaining permission, and the questionnaire was completed through phone calls.

### Measurements

#### Demographic information form

It contained questions about information related to age, gender, marital status, language, and residence.

#### Communication barrier questionnaire

The barriers to effective nurse-patient communication were investigated using the same questionnaire designed by Anoosheh et al. This questionnaire contains 30 items for nurses and aims to evaluate nurses’ views about the barriers to effective nurse-patient communication. The response of this questionnaire is in the Likert range (completely false = 1, false = 2, I have no opinion = 3, agree = 4, and completely agree = 5). The nurses’ questionnaire contains four dimensions, and the question numbers of each dimension include individual/social factors (1–8), occupational characteristics (9–17), patient’s clinical conditions (18–21), and environmental factors (22–30). The domain of individual/social factors includes questions such as the gender difference between the patient and the nurse, age difference, aggressiveness of nurses, etc. The domain of job characteristics includes questions about the high workload of nursing, the difficulty of nursing work, the low salaries of nurses, etc. The domain of the patient’s clinical condition also includes questions such as the severity of the disease, the presence of the patient’s companion, etc. The domain of environmental factors: where communication occurs is important. The nurse and the patient should feel calm and safe in the treatment environment. This domain also includes questions such as the Lack of facilities (welfare - treatment) for patients, the unsanitary condition of the patient’s room, the High cost of treating patients, etc. A pilot study was carried out to assess the face validity among nurses. In addition, the content validity was assessed by estimation of content validity ratio and content validity index among nursing educators. The internal consistency for the present questionnaire assessed by Cronbach’s alpha coefficient equal to 0.96 [25].

The patient questionnaire contains 15 questions and aims to evaluate the patients’ views about the barriers to effective nurse-patient communication. The response of this questionnaire is in the Likert range (completely false = 1, false = 2, I have no opinion = 3, agree = 4, and completely agree = 5). No separate dimension was considered for the patient questionnaire. The reliability based on internal consistency was reported using Cronbach’s alpha equal to 0.91 [25]. The total score of the questionnaire is obtained by summing up the total scores of all questions. The score of each dimension is obtained from the sum of scores for each question of that dimension. Higher scores in each dimension indicate the greater strength of that dimension as a barrier to effective nurse-patient communication and vice versa. After completing the communication barrier questionnaire, a separate question was asked from the patients and nurses about whether or not the face mask was a communication barrier. This question was scored with a Likert scale (completely false = 1, false = 2, I have no opinion = 3, agree = 4, and completely agree = 5). The score of this question was measured separately from the nurse-patient communication barrier questionnaire.

#### Ethical considerations

Initially, necessary permissions were obtained from the Vice Chancellor of Research and Technology and the Research Ethics Council (code of ethics: IR.SHMU.REC.1401.140) at the Shahroud University of Medical Sciences. Necessary coordination was also made with the administrators of two conveniently selected hospitals in Shahroud. After explaining the purpose of the research and answering the questions of nurses and patients regarding the questionnaire and how to complete them, enough time was given to answer them.

### Statistical analysis

Data were analyzed using descriptive statistics (frequency, percentage, mean, and standard deviation) and inferential tests (Linear regression) in SPSS software version 18. All variables with a significance level of less than 0.2 are included in the final regression model. A significance level of 0.05 was considered. Considering that one of the purposes of this study is to determine the barriers to effective nurse-patient communication based on demographic information, three participants were excluded from the data analysis due to a lack of demographic information completion.

## Results

The average ages of nurses and patients were respectively 33.28 and 38.57 years, and most nurses (85.3%) and patients (61.5%) were females and males, respectively. Other demographic characteristics are listed in Table 1.

**Table 1:** Demographic information of nurses and patients participating in the study

Variable		nurses		patients	
		N	%	N	%
**Gender**	**Male**	29	14.7	123	61.5
	**Female**	168	85.3	77	38.5
**Marital status**	**Married**	146	76.4	130	65.0
	**Single**	45	23.6	70	35.0
**Language**	**Persian**	197	100	192	96.0
	**Other languages**	-	-	8	4.0
**Residence**	**native**	170	87.2	144	73.8
	**non-native**	25	12.8	51	26.2
		**Mean**	**SD** ^*^	**Mean**	**SD**
**Age**		33.28	6.82	38.57	15.26

*SD: Standard Deviation;

In this study, the mean score obtained for each domain of the barriers to nurse-patient communication was determined from the nurses’ point of view. According to these results, the highest score with an average of 32.41 ± 6.75 related to the domain of job characteristics, and the lowest score with an average of 11.76 ± 3.17 related to the domain of Patient’s Clinical Conditions. Additional information is presented in Table 2.

**Table 2:** The mean score of barriers to effective communication among nurses in four domains

Domain	Mean	SD^*^
**Job Characteristics**	32.41	6.75
**Environmental Factors**	30.60	7.35
**Individual/Social Factors**	24.21	5.08
**Patient’s Clinical Conditions**	11.76	3.17
**Total**	98.99	16.77

*SD: Standard Deviation;

The excessive patients’ expectations in the domain of individual/social factors, the high workload of nursing in the domain of job characteristics, the severity of the disease in the domain of the patient’s clinical conditions, and no appreciation for nurses by authorities in the domain of environmental factors were the major communication barriers. The patient-nurse age difference from the domain of individual/social factors, the patient’s contact with multiple nurses with different attitudes from the domain of job characteristics, previous hospitalization history from the domain of the patient’s clinical conditions, and the high cost of patient treatment from the domain of environmental factors were the least important barriers to communication from the nurses’ viewpoints. From the patients’ views, the aggressiveness of nurses and the patient-nurse age difference were the major and the minor barriers to communication, respectively. Face masks were among the minor barriers to nurse-patient communication from the viewpoints of both groups (Table 3); this table is placed at the end of the article.

**Table 3:** Distribution of the frequency of nurses’ views regarding communication barriers

Domain	Mean (SD)	Barriers to nurse-patient communication	nurses		patients	
			Mean	SD^*^	Mean	SD
**Individual/Social Factors**	**3.02** **(0.63)**	Excessive expectations of patients	4.09	0.89	3.23	1.09
		The patient’s lack of familiarity with the description of nurses’ duties	3.58	1.15	3.20	1.18
		The aggressiveness of nurses	3.54	1.13	3.89	1.34
		The nurse’s lack of familiarity with the local language	3.49	1.04	2.96	1.05
		Problems outside of the nurses’ work environment	3.18	1.17	3.44	1.20
		The difference between the gender of the patient and the nurse	2.72	1.24	2.87	1.19
		The class difference between patient and nurse	2.19	1.10	2.66	1.15
		The age difference between the patient and the nurse	2.14	1.09	2.59	1.11
**Job Characteristics**	**3.60** **(0.75)**	The high workload of nursing	4.13	1.96	3.68	1.06
		The difficulty of nursing work	3.97	1.18	-	-
		The physical and mental fatigue of nurses	3.94	0.91	-	-
		Lack of welfare facilities for nurses	3.91	1.15	-	-
		The low salaries of nurses	3.76	1.30	-	-
		Nurses’ disinterest in their work	3.42	1.18	-	-
		Shift work of nursing	3.35	1.28	-	-
		Lack of information and skills of nurses in the field of communication with patients	3.34	1.10	-	-
		The patient’s contact with many nurses with different attitudes	3.29	1.05	3.16	1.05
**Patient’s Clinical Conditions**	**2.94** **(0.79)**	The severity of the disease	3.13	1.11	-	-
		companionship	3.10	1.13	2.63	1.36
		The patient suffering from infectious diseases	3.07	1.09	3.25	1.20
		Previous hospitalization history	2.75	1.10	2.68	1.01
**Environmental Factors**	**3.40** **(0.81)**	no appreciation for nurses by administrators	3.91	1.19	-	-
		Non-participation of nurses in the decisions of the work environment	3.82	2.37	-	-
		The feeling of injustice in the work environment	3.66	1.19	-	-
		Lack of facilities (welfare - treatment) for patients	3.61	2.34	3.72	1.17
		Failure to perform duties properly by other hospital staff	3.48	1.09	-	-
		Lack of teaching communication skills during the education of nurses	3.36	1.07	-	-
		Lack of training while serving communication skills	3.30	1.01	-	-
		The unsanitary condition of the patient’s room	3.00	1.09	3.70	1.33
		High cost of treating patients	2.97	1.10	-	-
Is the face mask considered a communication barrier?			2.77	1.25	2.52	1.09

*SD: Standard Deviation;

The relationship between nurses’ age and communication barriers was investigated using a regression model. This model was first run as a univariate type, and variables with a significance of < 0.2 were introduced into a multivariate model using the backward method. Finally, the model showed that the nurses’ age variable explained 3.8% of the score variance. In other words, the regression model revealed that the mean score of nurses would decrease to 0.486 for each year of age increase, and there is no statistically significant difference between the COVID and non-COVID wards (Table 4).

**Table 4:** The role of independent variables on communication barriers from the nurse’s point of view based on the regression model

Parameter	β	SE	t	p	95% Confidence Interval	
					Lower Bound	Upper Bound
**Intercept**	114.932	5.943	19.338	< 0.001	103.210	126.654
**Nurses’ age**	-0.486	0.175	-2.778	0.006	-0.831	-0.141
**COVID to Non-COVID ward**	1.087	2.989	0.364	0.717	-4.809	6.983

SE: Standard error; p: *P*-value;

Additionally, the patient’s residence variable explained 2.3% of the score variance, meaning that native people obtained a mean score of 2.813 units less than non-native people, and there is no statistically significant difference between the COVID and non-COVID wards (Table 5).

**Table 5:** Assessment of the predictive role of independent variables on communication barriers from the patient’s view point based on the regression model

Parameter	β	SE	t	p	95% Confidence Interval	
					Lower Bound	Upper Bound
**Intercept**	49.000	1.142	42.919	< 0.001	46.748	51.252
**Native**	-2.813	1.329	-2.117	0.036	-5.433	-0.192
**Non-Native**	Reference	-	-	-	-	-
**COVID ward**	0.463	1.390	0.333	0.740	-2.278	3.204
**Non-COVID ward**	Reference					

SE: Standard error; p: *P*-value;

## Discussion

The present study aimed to determine the barriers to effective nurse-patient communication from the viewpoints of nurses and patients in COVID-19 and non-COVID-19 wards in hospitals affiliated with the Shahroud University of Medical Sciences. The results of this study showed that in the domains of barriers to effective communication, nurses reported the highest score in job characteristics and the lowest score in the patient’s clinical conditions. In a study on nursing students at Urmia Midwifery School of Nursing, Habibzadeh et al. (2017) reported the highest and the lowest mean scores for questions related to occupational characteristics and the patient’s clinical conditions [26], which corresponds to our results. Work congestion conditions increase the work pressure of nurses, leading to fatigue, a situation in which nurses lack enough time to discover the patient’s concerns [27]. Stress and pressure caused by time constraints often result in miscommunication and reduce the satisfaction of nurses and patients [28].

The results of this study showed that the high workload of nursing and excessive expectations of patients are mentioned as two major obstacles to effective communication with patients from the point of view of nurses. Anoushe et al. (2015) and Baraz Pordanjani et al. (2016) investigated barriers to effective nurse-patient communication. They reported that nurses identify their workload as a major barrier to effective patient communication [15, 22]. However, Habibzadeh et al. (2017) claimed that nurses’ lack of information and skills in patient communication was identified as the main communication barrier [26]. A possible reason for this discrepancy might be that the current study was conducted during the COVID-19 pandemic, concurrent with the increased workload of nurses compared to the pre-pandemic period.

The difficulty of nursing work, the psychophysical fatigue of nurses, the lack of comfort facilities for nurses, and no appreciation for nurses by administrators are in the next ranks of importance. Similarly, Anoushe et al. (2005) reported the difficulty of nursing work, the lack of comfort facilities for nurses, and psychophysical fatigue among the barriers with more emphasis by nurses [22]. The notable point is that nurses do not have the opportunity to establish effective communication with patients due to their workload. Furthermore, their work type is hard and tiring, and they do not receive proper benefits or appreciation. In such a situation, one cannot expect good nurse-patient communication, and the conditions affect patients’ moods. As expressed by the patients, this issue also negatively affects the quality of their relationships with patients [15].

The aggressiveness of nurses mentioned as the main obstacle to effective communication with patients from the patients’ point of view. Likewise, Baraz Pordanjani et al. (2009) found a statistically significant difference between the aggressiveness of nurses from the perspectives of nurses and patients [15].

Regarding the communication barriers from the patient’s perspective, the lack of facilities (welfare treatment) for them and the unsanitary condition of their rooms were among the factors more emphasized by patients than by the nurses. Interestingly, Baraz Pordanjani et al. observed that nurses believed more than patients that the lack of comfort facilities for patients and the unsanitary condition of their rooms would hinder effective communication [15]. This contradictory result can result from the difference in facilities and health/treatment conditions of the studied hospitals.

The viewpoints of both nurse and patient groups show that age and class differences do not negatively influence their relationships. Since nurses are responsible for initiating and maintaining communication with patients, it can be claimed that they perform their professional tasks, including communication establishment, regardless of the social class and age of patients, who also acknowledge this issue.

The face mask also obtained a low score from the viewpoints of patients and nurses. Vitale et al. investigated the use of face masks as a communication barrier between nurses and patients. The results indicated no difference in the patients’ opinions before and during the COVID-19 pandemic; that is, patients did not consider the mask a communication barrier, which is consistent with the present study. However, nurses thought that using a mask would be a communication barrier [12].

The present results revealed a significant relationship between the age of nurses and the barriers to effective nurse-patient communication; as such, the total score of nurses decreased for each year of age increase; However, no statistically significant difference was observed in the comparison of COVID and non-COVID wards. In this regard, Gopichandran et al. (2021) aimed to determine communication barriers between doctors and patients during the COVID-19 pandemic in India. They claimed that communication barriers decreased with age [29]. Nurses gain more experience and skills with rising age. Enough experience is also a characteristic that patients consider necessary for nursing work [30]. “According to Aram Feizi et al. (2006)” Mark (2001) concluded that the experience of the nursing unit could create satisfaction in both nurses and patients [30]. The possible reason for obtaining different results could be that the COVID-19 vaccination process was carried out slowly in Iran. For this reason, the nurses, both in the COVID and non-COVID wards, considered all patients with unique viewpoints (all of the patients considered potential cases of COVID-19). For this reason, there was no statistical difference between the communication barriers of the COVID and non-COVID departments.

No statistically significant difference was observed between the scores of male and female nurses and the barriers to effective nurse-patient communication. Unlike this result, Mohammadi et al. (2013) reported a significant difference between job characteristics, patients’ clinical conditions, environmental factors, and the gender of nurses [23]. The discrepant results might be caused by the heterogeneous distribution of participants in terms of gender, as 56% of the nurses were male in the study of Mohammadi et al. In comparison, less than 20% of the participants were male nurses in the present study.

The present results showed that the patients’ residence was significantly related to the barriers to effective nurse-patient communication, and native people obtained a lower mean score than non-native people: However, there was no any no significant difference between COVID and NON-COVID wards This result might be because nurses are more informed of the accents and dialects of native patients. Caring for patients speaking different languages and accents can lead to problems in the quantity and quality of nurse-patient communication. When patients and caregivers have different cultural values and languages, communication can cause the inability to exchange information [27]. Tilki and Okoughan presented evidence that differences in spoken language could hinder effective communication [31]. On the other hand, the results of the study by Vitale et al. showed that there was no difference between the patients before and during the covid-19 pandemic, which is consistent with the results of the present study [12].

### Limitations

Among the limitations of this study, we can mention the low response rate by nurses and patients, which was completed with the continuous presence of the researcher. Since this research is conducted only in public medical centers affiliated to Shahroud University of Medical Sciences, the results may not be generalizable to centers affiliated with other universities of medical sciences in Iran and non-academic centers such as private medical centers. It is recommended that future research be conducted in larger settings.

## Conclusion

This study demonstrated that nurses identified the domain of job characteristics as the most critical barrier among the four domains of barriers to effective nurse-patient communication. Patients more emphasized factors that were in the domain of individual/social factors. There is a pressing need to pay attention to these barriers to eliminate them through necessary measures by nursing officials. Hopefully, the elimination of these barriers in the future will lead to nurses who can communicate well with patients and improve service delivery.

### Implications

This research helps to identify barriers to effective communication between nurses and patients. In the field of policy and management, the results of this research can help to plan for effective nurse-patient communication. In the field of education, according to the results of this article, necessary training should be given to nurses and patients regarding communication barriers to help improve communication. There will be a basis for further, more comprehensive research in the field of research. Hopefully, these results can help nursing officials and nurses remove communication barriers and improve service delivery.

## Data Availability

The datasets used and/or analysed during the current study available from the corresponding author on reasonable request.
